# Population-Based Study of Pertussis Incidence and Risk Factors among Persons >50 Years of Age, Australia

**DOI:** 10.3201/eid3001.230261

**Published:** 2024-01

**Authors:** Rodney Pearce, Jing Chen, Ken L. Chin, Adrienne Guignard, Leah-Anne Latorre, C. Raina MacIntyre, Brittany Schoeninger, Sumitra Shantakumar

**Affiliations:** Medical HQ Family Practice, Glynde, South Australia, Australia (R. Pearce); GSK, Singapore (J. Chen, S. Shantakumar);; IQVIA, Melbourne, Victoria, Australia (K.L. Chin);; Monash University, Melbourne (K.L. Chin);; GSK, Wavre, Belgium (A. Guignard); GSK, Melbourne (L.-A. Latorre);; University of New South Wales, Sydney, New South Wales, Australia (C.R. MacIntyre); IQVIA, Sydney (B. Schoeninger)

**Keywords:** Pertussis, incidence, Australia, risk factors, vaccine-preventable diseases, vaccination, elderly, whooping cough, asthma, COPD, bacteria, respiratory infections

## Abstract

Despite vaccination programs, pertussis has been poorly controlled, especially among older adults in Australia. This longitudinal, retrospective, observational study aimed to estimate the incidence and risk factors of pertussis among persons ≥50 years of age in Australia in the primary care setting, including those with underlying chronic obstructive pulmonary disease (COPD) or asthma. We used the IQVIA general practitioner electronic medical record database to identify patients ≥50 years of age with a clinical diagnosis of pertussis during 2015–2019. Pertussis incidence rates ranged from 57.6 to 91.4 per 100,000 persons and were higher among women and highest in those 50–64 years of age. Patients with COPD or asthma had higher incidence rates and an increased risk for pertussis compared with the overall population ≥50 years of age. Our findings suggest that persons ≥50 years of age in Australia with COPD or asthma have a higher incidence of and risk for pertussis diagnosis.

Pertussis, or whooping cough, a bacterial respiratory infection caused by *Bordetella pertussis*, is typically characterized by paroxysms of coughing with a whooping sound during inhalation. In Australia, pertussis is one of the least well-controlled vaccine-preventable diseases (*1*). Robust childhood and maternal immunization programs prevent pertussis-related hospitalizations and mortality in infants and children, but breakthrough disease can occur at any age, because naturally acquired and vaccine-induced immunity against pertussis is not lifelong (*2*,*3*). Although older adults can be a source of transmission and adversely affected by infection, vaccination programs are prioritized for pregnant women (preferably 20–32 weeks’ gestation), infants and children (i.e., ages 2, 4, 6 and 18 months, and 4 years), and adolescents (11–13 years of age) (*1*). Consequently, pertussis in older adults constitutes a considerable reservoir of infection and contributes to substantial disease burden, healthcare utilization, and other pertussis-associated costs (*1*).

Recent research has offered a clearer assessment of pertussis cases among adults ≥50 years of age (*4*). In Australia, pertussis-containing (i.e., combined diphtheria, tetanus, and acellular pertussis) vaccines are recommended for adults at 50 years and 65 years of age by the Australian Technical Advisory Group on Immunisation (*1*,*5*,*6*) but are not funded by the National Immunisation Program. Both confirmed and probable cases of pertussis must be reported to the Commonwealth’s National Notifiable Diseases Surveillance System (NNDSS) (*5*). However, pertussis infections are often underdiagnosed and underreported, owing to difficulties in diagnosis because of unspecific symptoms, delays in seeking healthcare, underuse of diagnostic testing by general practitioners (GPs), and other coexisting respiratory diseases. Accordingly, NNDSS notification data tend to underestimate the true number of pertussis infections and are more likely to capture persons with severe disease (*7*), posing serious challenges to policy considerations for pertussis vaccination in adults ≥50 years of age. Given the underuse of diagnostic testing by GPs, pertussis diagnoses are based frequently on clinical evidence. Beyond NNDSS data, alternative sources of data, including electronic medical records (EMRs), may inform understanding of the burden of pertussis in persons ≥50 years of age in Australia (hereafter Australians >50 years) in the primary care setting.

Patients with underlying respiratory conditions, such as asthma and chronic obstructive pulmonary disease (COPD), have an increased risk for pertussis, leading to more severe outcomes and exacerbation of asthma and COPD (*8*–*10*). The considerable overlap in the clinical manifestations of those conditions can complicate the diagnosis of pertussis in such patients. In this study, we estimated the incidence rate (IR) of pertussis based on clinical diagnoses and determined the risk factors of pertussis among Australians ≥50 years, including those with COPD or asthma, using the IQVIA GP EMR database.

## Methods

### Data Sources

We conducted a longitudinal, retrospective, observational study by using records in the IQVIA GP EMR database dated January 2015–December 2019. The IQVIA GP EMR database contains de-identified patient records collected from the EMR software of consenting GPs in Australia, covering 2,286,308 patients across 2,500 GPs and averaging 6 years’ follow-up per patient. The database provides rich data that includes demographic and biometric profiles, medical profiles, prescribing information, and prescriber characteristics.

### Study Population

**Figure 1:**
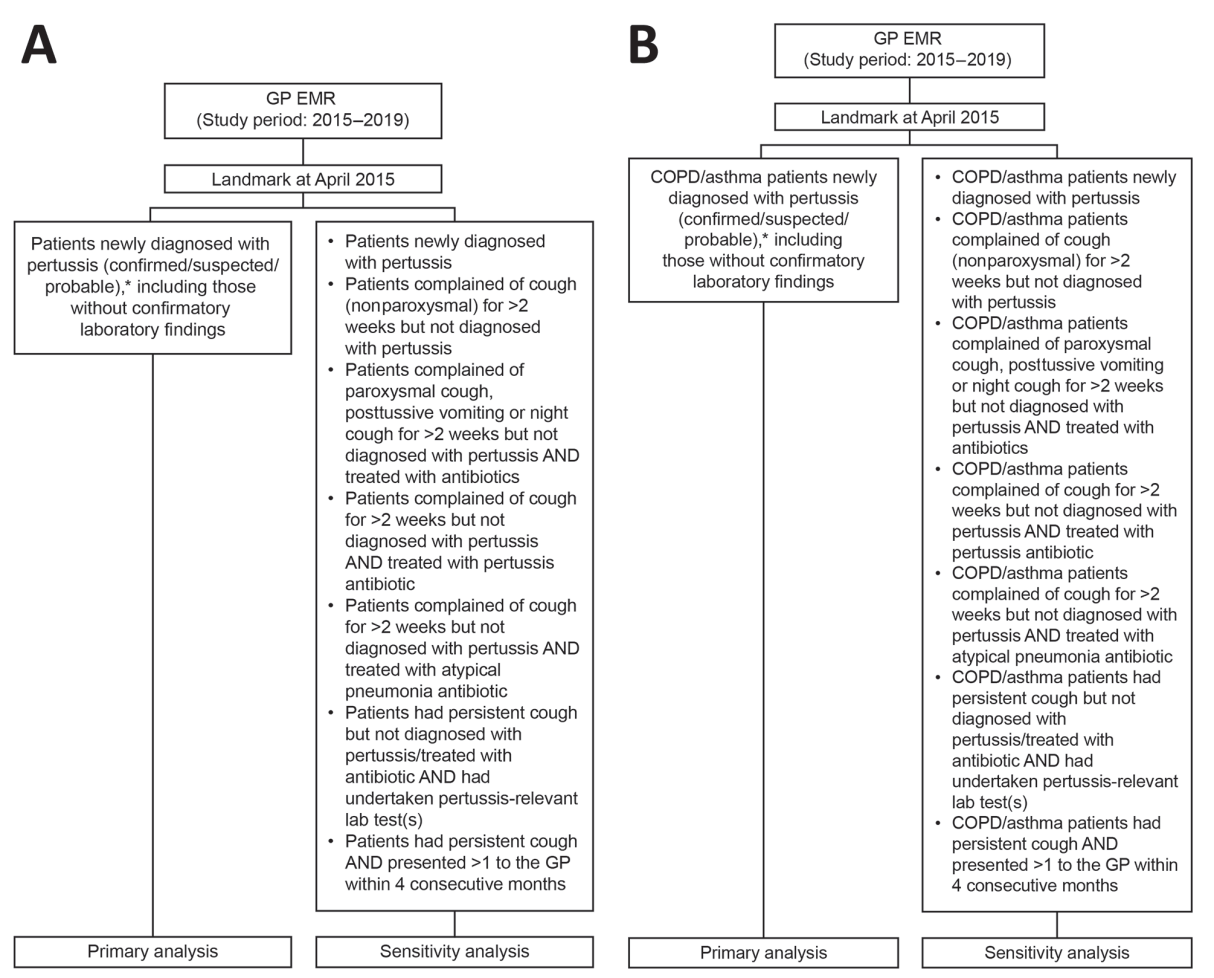
Identification of pertussis cases in population-based study of pertussis incidence and risk factors among persons >50 years of age, Australia. A) Overall study population; B) patients with COPD/asthma. *Differentiation between confirmed, probable, and suspected cases was not possible because lab testing is not routinely being performed in all patients and cases were identified from the GP EMR based on the diagnosis label. COPD, chronic obstructive pulmonary disease; EMR, electronic medical record; GP, general practitioner.

The study population included patients ≥50 years of age whose records were collected in the IQVIA GP EMR database during January 2015–December 2019, with pertussis as the outcome of interest. We identified pertussis cases in the overall study population and in patients with COPD or asthma (Figure 1). We used search terms to identify pertussis, COPD, and asthma cases for the primary and sensitivity analyses (Appendix Table 1). We performed sensitivity analyses on a secondary patient population, applying a broader pertussis definition to assess the robustness of estimates, including patients with prolonged coughing potentially related to undiagnosed pertussis, treated empirically as atypical pneumonia or upper respiratory tract infections (Appendix Table 2).

We excluded patients with a diagnosis of pertussis before April 30, 2015, because a run-in period was required to rule out prevalent cases carried forward from the end of 2014. We also excluded patients without a history of diagnosed pertussis during the study period who visited their GP for pertussis vaccination or travel immunization.

### Study Objectives

The primary objective of our study was to estimate pertussis incidence based on clinical evidence captured in the IQVIA GP EMR database in Australians ≥50 years, including those with COPD or asthma. The secondary objective was to determine the risk factors for pertussis in this population.

### Data Analysis

We calculated the IR of pertussis (cases/100,000 persons) in the overall study population and in patients with COPD or asthma per calendar year as the number of new cases of pertussis divided by the corresponding population in the dataset for each year. We parametrically estimated IRs with 95% CIs by fitting Poisson or negative binomial distributions as appropriate. We compared qualitative or discrete variables by using Pearson χ^2^ or Fisher exact tests, and we compared quantitative variables by using 2-sample Student t-test or analysis of variance, after checking equality of variance (Fisher test) and normality (Shapiro–Wilk test). We used Wilcoxon, Mann–Whitney, or Kruskal–Wallis tests if equality of variance and normality were not confirmed.

To determine risk factors for pertussis, we compared patients with diagnosed pertussis with controls by using a nested case-control design. We defined controls as patients who had never received a diagnosis of pertussis during the study period. We matched case patients and controls based on age group (in 5-year groups >50 years of age), sex, postal code, and year of GP visit, by using a 1:3 ratio within the same calendar year. We used conditional logistic regression to estimate odds ratios (ORs) and 95% CIs. We examined all exposure variables for univariate associations and constructed a multivariable model that included age, sex, and any variables with p value ≤0.2 in univariate analyses. We also performed multivariable logistic regressions based on forward and backward elimination methods to confirm robustness of the estimates. Given its association with pertussis diagnoses in a previous report, asthma was retained in the final model a priori, irrespective of statistical significance (*11*). Likewise, use of angiotensin-converting enzyme inhibitors (known to induce cough) or angiotensin receptor blockers (known to protect against pertussis infection) and history of pertussis vaccination (during the study period) were retained in the final model a priori, irrespective of statistical significance (*4*,*12*). We performed additional exploratory analyses to evaluate health-seeking behavior by estimating the number of GP visits per year before pertussis diagnosis and antibiotic prescriptions for cough 1 month before pertussis diagnosis; we then further stratified these data by age, sex, and underlying COPD or asthma. We performed all analyses by using R version 4.3 (The R Foundation for Statistical Computing, https://www.r-project.org).

## Results

### Study Population

The total population of Australians ≥50 years in the IQVIA GP EMR database covered 252,227–415,201 patients for each year during 2015–2019. We selected 992 patients with pertussis, including those not tested for pertussis, for inclusion in the primary analysis based on the diagnosis label (Table 1). Laboratory tests for pertussis were ordered for 243 (24%) patients and results documented for 205 (21%) patients, of which only 10 (0.01%) tested positive for pertussis. Because of the limited diagnostic testing performed for patients with diagnosed pertussis in GP clinics, our study relied instead on the clinical diagnosis of pertussis.

**Table 1:** Baseline patient characteristics for persons ≥50 years of age with pertussis identified in the overall study population, Australia*

Characteristics	Year						p value†
	2015, n = 253	2016, n = 251	2017, n = 219	2018, n = 162	2019, n = 148	Total, n = 992	
Age, y							
Mean (95% CI)	62.5 (61.3–63.7)	63.1 (61.9–64.3)	63.1 (61.9–64.2)	65 (63.4–66.5)	63.8 (62.3–65.4)	63.3 (62.7–63.9)	0.22
Median (IQR)	61 (54–68)	61 (54–68)	63 (56.5–69.5)	63 (56–70)	63 (55.6–70.5)	62 (55.5–68.5)	0.15
Sex‡							
M	91 (36)	77 (31)	73 (33)	56 (35)	49 (33)	332 (33)	0.61
F	162 (64)	173 (69)	146 (67)	105 (65)	99 (67)	658 (67)	0.61
Smoking status							
Never	101 (41)	107 (45)	94 (44)	67 (41)	70 (47)	433 (44)	0.61
Past	49 (20)	45 (19)	53 (25)	51 (31)	33 (22)	216 (22)	0.46
Current	15 (6)	22 (9)	17 (8)	14 (9)	9 (6)	75 (8)	0.61
Unknown	79 (32)	63 (27)	51 (24)	30 (19)	36 (24)	268 (27)	0.13
Alcohol consumption/d§							
None	94 (39)	97 (41)	86 (40)	75 (46)	75 (51)	426 (43)	0.09
≤1 unit	39 (16)	32 (14)	35 (16)	28 (17)	28 (19)	161 (16)	0.13
>1 unit	18 (7)	12 (5)	14 (7)	15 (9)	18 (12)	76 (8)	0.13
Unknown	93 (38)	96 (41)	80 (37)	44 (27)	27 (18)	329 (33)	0.09
Vaccination history¶							
Pertussis	36 (14)	29 (12)	36 (16)	31 (19)	25 (17)	140 (14)	0.22
Influenza	2 (1)	49 (20)	68 (31)	71 (44)	59 (40)	241 (24)	0.09
Pneumococcal	8 (3)	11 (4)	15 (7)	11 (7)	21 (14)	60 (7)	0.04
Residence							
New South Wales	83 (34)	54 (23)	51 (24)	34 (21)	49 (33)	282 (29)	0.81
Victoria	64 (26)	78 (33)	71 (33)	40 (25)	29 (20)	258 (26)	0.31
Queensland	29 (12)	26 (11)	27 (13)	15 (9)	9 (6)	111 (11)	0.22
Western Australia	21 (9)	17 (7)	15 (7)	12 (7)	7 (5)	70 (7)	0.10
South Australia	12 (5)	10 (4)	6 (3)	3 (2)	6 (4)	38 (4)	0.31
Tasmania	5 (2)	11 (5)	12 (6)	35 (22)	30 (20)	89 (9)	0.09
Australian Capital Territory	26 (11)	31 (13)	23 (11)	14 (9)	11 (7)	105 (11)	0.13
Northern Territory	4 (2)	10 (4)	10 (5)	9 (6)	7 (5)	39 (4)	0.13
Comorbidities#							
COPD	41 (16)	51 (20)	40 (18)	38 (23)	25 (17)	185 (19)	0.81
Asthma	25 (10)	35 (14)	27 (12)	24 (15)	16 (11)	117 (12)	0.81
CVD	101 (40)	111 (44)	96 (44)	78 (48)	57 (39)	409 (41)	0.61
Heart failure	4 (2)	4 (2)	3 (1)	2 (1)	9 (6)	16 (2)	0.61
Diabetes mellitus	32 (13)	32 (13)	36 (16)	34 (21)	18 (12)	144 (14)	0.61
History of stroke	3 (1)	3 (1)	3 (2)	2 (1)	9 (6)	21 (2)	0.27
Chronic kidney disease	5 (2)	4 (2)	10 (5)	4 (2)	5 (3)	27 (3)	0.58
Cancer, active or remission	23 (9)	45 (18)	32 (15)	29 (18)	22 (15)	136 (14)	0.79

*Values are no. (%) except as indicated. Variables were defined as those at baseline (i.e., restricted to those recorded during the study period or until the time of pertussis diagnosis). COPD, chronic obstructive pulmonary disease; CVD, cardiovascular disease (except heart failure); IQR, interquartile range. †Pearson χ^2^ or Fisher exact test for categorical variables, Student t-test/Wilcoxon/Kruskal Wallis tests for continuous variables, as appropriate. There were significant differences in smoking status, alcohol consumption, state of residence (Western Australia, Australian Capital Territory), COPD, and diabetes between males and females. ‡Two patients had sex documented as unknown. §One unit of alcohol is defined as containing 10 g of alcohol according to Australia’s national alcohol guidelines. ¶History of pertussis immunization was defined as the presence of pertussis immunization record throughout the study period, as recorded in the Australia general practitioner electronic medical records database. #History of CVD, chronic kidney disease, and cancer were defined as those at baseline and were restricted to those recorded during the study period, or until the time of pertussis diagnosis, as applicable.

Among 992 patients with diagnosed pertussis, 66% were women, 41% had a history of cardiovascular disease, 19% had COPD, and 12% had asthma; mean age was 63.3 (95% CI 62.7–63.9) years. Fourteen percent of patients had a record of pertussis immunization during the study period and before pertussis diagnosis. A total of 2,543 patients with pertussis met the extended definition of pertussis for the sensitivity analysis (Appendix Tables 3, 4).

### Incidence of Pertussis

**Figure 2:**
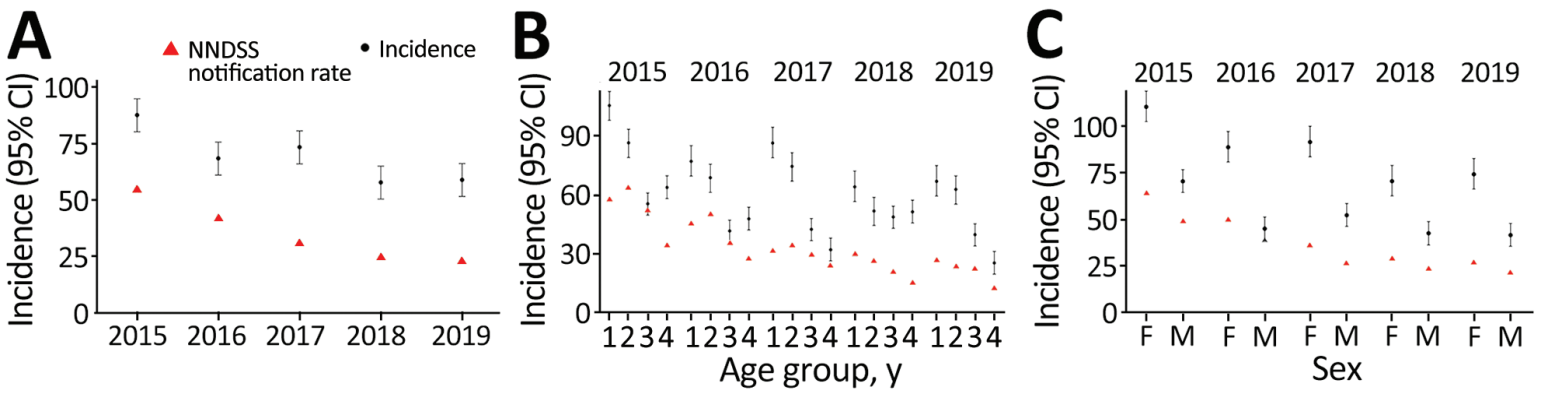
National notification rates compared with annual incidence of pertussis in population-based study of pertussis incidence and risk factors among persons >50 years of age, Australia. A) Overall study population; B) by age group: group 1, 50–64 y; group 2, 65–74 y; group 3, 75–84 y; group 4, ≥85 y; C) by sex. Incidence rates are reported per 100,000 persons; error bars indicate 95% CIs. Both NNDSS and GP EMR data consist of persons ≥50 years of age. Data in 2015 were projected to 12-month period because a run-in period/landmark was applied to rule out prevalent pertussis cases carried forward from the previous year. Data for 2016–2019 are as observed. EMR, electronic medical records; GP, general practitioner; NNDSS, National Notifiable Diseases Surveillance System.

We observed differences in annual incidence of pertussis among Australians ≥50 years in 2015–2019 (Figure 2, panel A; Appendix Table 5). Pertussis IRs per 100,000 persons were 91.4 (95% CI 84.1–98.7) in 2015 and 58.7 (95% CI 51.4–66.0) in 2019 (Figure 2, panel A). In age subgroups, IRs were consistently highest among those 50–64 years of age and 65–74 years of age (Figure 2, panel B; Appendix Table 5). Overall, pertussis IRs varied across all age groups during 2015–2019 (Figure 2, panel B).

When stratified by sex, pertussis IRs among women were 110.5 (95% CI 102.3–118.7) in 2015 and 74.5 (95% CI 66.3–82.7) in 2019. Lower IRs of 70.6 (95% CI 64.3–76.8) in 2015 and 41.6 (95% CI 35.4–47.8) in 2019 were observed among men (Figure 2, panel C). Similar trends were reported by the NNDSS, although pertussis IRs were lower than those in our study population (Figure 2).

**Figure 3:**
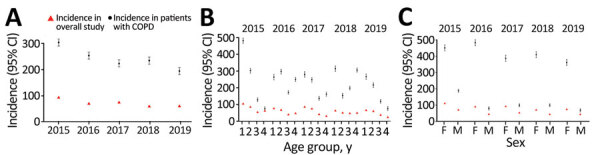
Annual incidence of pertussis among persons >50 years of age with and without COPD in population-based study of pertussis incidence and risk factors, Australia. A) Overall study population; B) by age group: group 1, 50–64 y; group 2, 65–74 y; group 3, 75–84 y; group 4, ≥85 y; C) by sex. Incidence rates are reported per 100,000 persons; error bars indicate 95% CIs. COPD cases were defined based on diagnosis label or prescription of reliever/corticosteroid inhaler (≥1 refill of the same product). Data in 2015 were projected to 12-month period because a run-in period/landmark was applied to rule out prevalent pertussis cases carried forward from the previous year. Data for 2016–2019 are as observed. COPD, chronic obstructive pulmonary disease.

**Figure 4:**
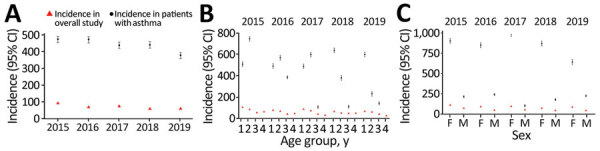
Annual incidence of pertussis among persons >50 years of age with and without asthma in population-based study of pertussis incidence and risk factors, Australia. A) Overall study population; B) by age group: group 1, 50–64 y; group 2, 65–74 y; group 3, 75–84 y; group 4, ≥85 y; C) by sex. Incidence rates are reported per 100,000 persons; error bars indicate 95% CIs. Asthma cases were defined based on diagnosis label or prescription of reliever/corticosteroid inhaler (≥1 refill of the same product). Data in 2015 were projected to 12-month period because a run-in period/landmark was applied to rule out prevalent pertussis cases carried forward from the previous year. Data for 2016–2019 are as observed.

In patients with COPD, pertussis IRs throughout the study period ranged from 304.4 (95% CI 290.7–318.1) in 2015 to 194.3 (95% CI 180.5–207.9) in 2019 (Figure 3, panel A; Appendix Table 6). Similarly, in patients with asthma, pertussis IRs ranged from 473.0 (95% CI 454.0–492.0) in 2015 to 376.8 (95% CI 357.8–395.8) in 2019 (Figure 4, panel A; Appendix Table 7). Although pertussis IRs among patients with COPD or asthma were higher than the overall study population, variations between age groups were generally consistent (Figure 3, panel B; Figure 4, panel B). Consistent with the overall study population, pertussis IRs in women with COPD or asthma were higher than in men with COPD (Figure 3, panel C; Appendix Table 6) or in men with asthma (Figure 4, panel C; Appendix Table 7).

**Figure 5:**
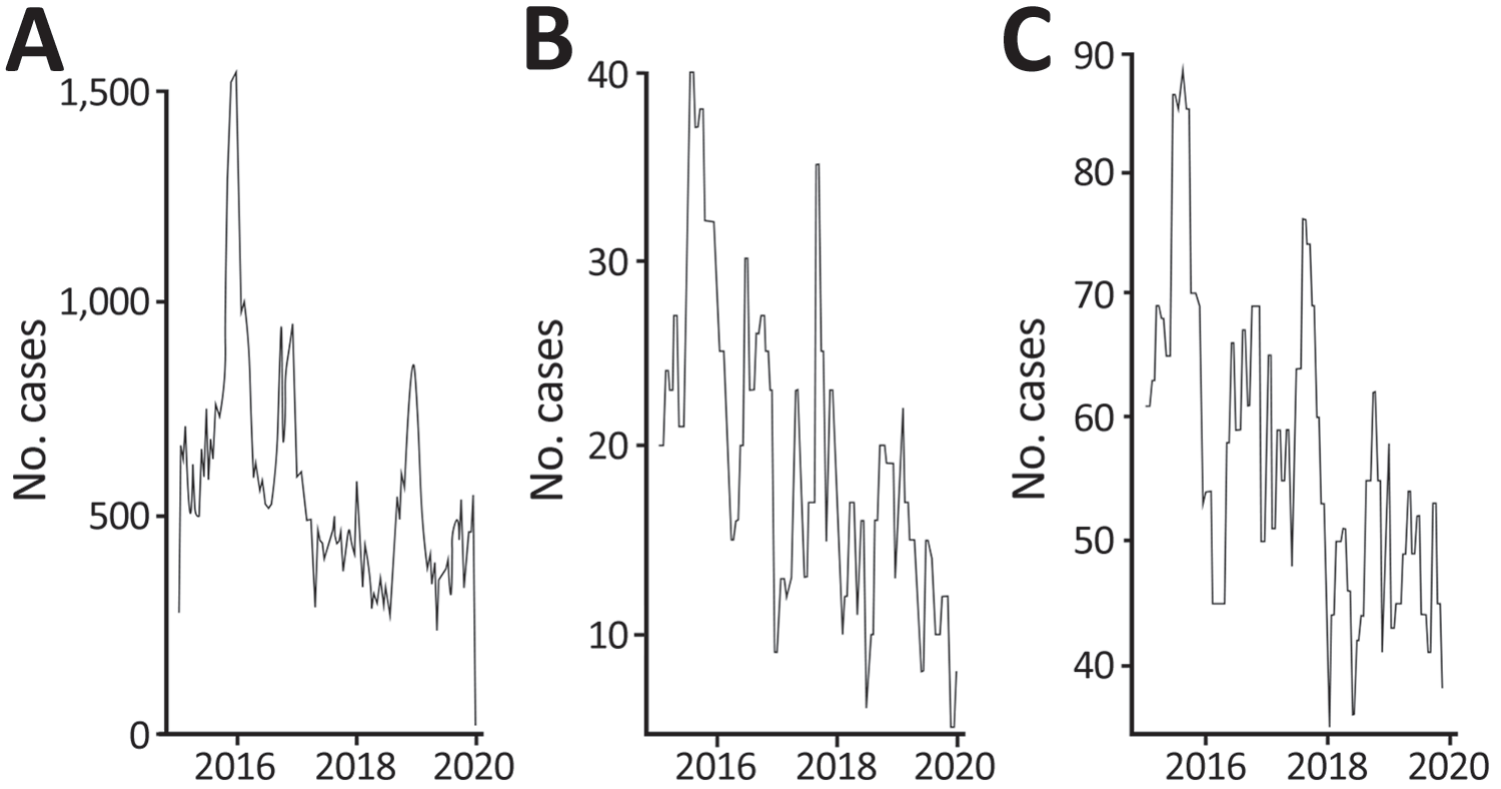
Comparison of the monthly number of pertussis cases reported to the NNDSS and identified in the primary and sensitivity analyses of population-based study of pertussis incidence and risk factors among persons >50 years of age, Australia. A) Cases captured by the NNDSS; B) cases in the primary overall study population; C) cases in the sensitivity analysis cohort. NNDSS, National Notifiable Diseases Surveillance System.

In the sensitivity analysis, based on an extended definition of pertussis, annual IRs were 197.6 (95% CI 185.5–209.7) in 2015 and 196.7 (95% CI 184.5–208.8) in 2019 (Appendix Figure 1, panel A). Overall, the lowest IRs were observed in patients ≥85 years of age (Appendix Figure 1, panel B). IRs remained higher among women versus men (Appendix Figure 1, panel C). In the sensitivity analysis, IRs among patients with COPD or asthma were higher than those in the overall study population (Appendix Figure 2, panel A, Figure 3, panel A). In 2016–2018, pertussis IRs in patients with COPD or asthma were highest in patients 65–74 years of age (Appendix Figure 2, panel B, Figure 3, panel B). Overall, IRs by age group and sex in patients with COPD or asthma were higher than IRs for the same group in the primary analysis and NNDSS, consistent with trends observed in the primary analysis (Appendix Figure 2, panels B, C, Figure 3, panels B, C). Trends observed in the monthly number of pertussis cases defined within the primary and sensitivity analyses cohorts and those reported in the NNDSS data were generally consistent and peaked in winter (June–September) (Figure 5).

### Risk Factor Modeling

Conditional univariable logistic regression models predicted that concurrent diabetes, asthma, or COPD, use of angiotensin-converting enzyme inhibitors or angiotensin receptor blockers, and prior use of antibiotics within 1 month increased the risk for a pertussis diagnosis; history of influenza vaccination was associated with reduced risk for a pertussis diagnosis (Table 2). The adjusted multivariable logistic regression model suggested that prior prescription of an antibiotic for cough within 1 month (OR 7.00 [95% CI 4.21–11.64]), asthma (OR 2.94 [95% CI 1.71–5.04]), and COPD (OR 1.88 [95% CI 1.23–2.86]) were predictors of pertussis diagnosis, but the risk for pertussis diagnosis was lower for patients with a history of influenza vaccination (OR 0.38 [95% CI 0.26–0.55]; Table 3).

**Table 2:** Predictors of pertussis in persons ≥50 years of age from conditional univariable logistic regression models in the overall study population, Australia*

Variable	OR (95% CI)	p value
Smoking status		
No	Referent	
Yes	1.18 (0.88–1.58)	0.80
Alcohol consumption/d†		
None	Referent	
≤1 unit	1.02 (0.71–1.46)	0.93
>1 unit	1.04 (0.67–1.63)	0.86
Immunization history‡		
Pertussis	0.77 (0.48–1.24)	0.28
Influenza	0.52 (0.37–0.73)	<0.001
Pneumococcal	0.70 (0.41–1.17)	0.17
Comorbidities§		
CVD	1.30 (0.97–1.74)	0.08
Heart failure	0.93 (0.33–2.63)	0.90
Diabetes mellitus	1.65 (1.13–2.42)	<0.001
Stroke	0.88 (0.32–2.42)	0.80
Chronic kidney disease	0.90 (0.42–1.92)	0.78
Cancer	1.45 (0.98–2.15)	0.06
Asthma	2.46 (1.54–3.93)	<0.001
COPD	2.01 (1.38–2.93)	<0.001
Asthma/COPD	2.2 (1.59–3.05)	<0.001
Use of ACEi-ARB	1.45 (1.09–1.93)	<0.001
Prior use of antibiotics	7.19 (4.42–11.69)	<0.001

*Variables were defined as those at baseline (i.e., restricted to those recorded during the study period or until the time of pertussis diagnosis). In this analysis, patients with missing data were excluded. ACEi-ARB, angiotensin-converting enzyme inhibitors–angiotensin receptor blockers; COPD, chronic obstructive pulmonary disease; CVD, cardiovascular disease (except heart failure); OR, odds ratio. †One unit of alcohol is defined as containing 10 g of alcohol. ‡History of pertussis immunization was defined as the presence of pertussis immunization record throughout the study period, as recorded in the Australia general practitioner electronic medical records database. §History of CVD, chronic kidney disease, and cancer were defined as those at baseline and were restricted to those recorded during the study period, or until the time of pertussis diagnosis, as applicable.

**Table 3:** Predictors of pertussis in persons ≥50 years of age from conditional multivariable logistic regression models, Australia*

Variables	aOR (95%CI)
Prior prescription of antibiotics for cough	7.00 (4.21–11.64)
Asthma	2.94 (1.71–5.04)
COPD	1.88 (1.23–2.86)
Diabetes mellitus	1.47 (0.68–2.27)
Use of ACEi-ARB	1.37 (1.00–1.85)
History of pertussis immunization†	1.03 (0.62–1.69)
History of influenza vaccination	0.38 (0.26–0.55)

*Variables were defined as those at baseline (i.e., restricted to those recorded during the study period or until the time of pertussis diagnosis). In this analysis, patients with missing data were excluded. ACEi-ARB, angiotensin-converting enzyme inhibitors–angiotensin receptor blockers; COPD, chronic obstructive pulmonary disease. †History of pertussis immunization was defined as the presence of pertussis immunization record throughout the study period, as recorded in the Australia general practitioner electronic medical records database.

### Healthcare-Seeking Behavior and Antibiotic Prescriptions 

We compared the number of GP visits for men and women in terms of asthma status, COPD status, and across age groups (Appendix Figure 4, panel A). We observed no sex-specific differences (p = 0.12) among patients with COPD or asthma (women, median 11, interquartile range [IQR] 10, vs. men, median 10, IQR 13).

In the overall study population, the median number of GP visits within 1 year before pertussis diagnosis was significantly higher (p = 0.001) for women (median 8, IQR 10) than for men (median 6, IQR 9). The median number of GP visits increased with presence of COPD or asthma for both women and men. In patients without COPD or asthma, the median number of GP visits was also significantly higher (p = 0.01) in women (median 6, IQR 8) compared with men (median 5, IQR 9). Furthermore, the number of GP visits increased with age, independent of sex and COPD or asthma status (Appendix Figure 4, panels B, C).

To investigate the observed reduced risk for pertussis associated with history of influenza vaccination within the risk factor modeling, we also looked at the distributed number of GP visits among those with or without history of influenza vaccination before pertussis diagnosis. Persons vaccinated for influenza before pertussis diagnosis had a higher median number of GP visits compared with those without influenza vaccination across all age groups (Table 4).

**Table 4:** Comparison of median number of GP visits within 1 year before pertussis diagnosis by history of influenza vaccination and age groups in persons ≥50 years of age, Australia*

Age group, y	History of influenza vaccination, median (IQR) visits		p value†
	No	Yes	
50–64	5 (8)	9 (8)	<0.001
65–74	6 (9.5)	13 (9)	<0.001
75–84	8 (12)	13 (15)	<0.001
≥85	8 (10.5)	15 (17)	0.01

*GP, general practitioner; IQR, interquartile range. †Differences in median number of GP visits within 1 year before the pertussis diagnosis within each age group.

Overall, 252 (25%) patients diagnosed with pertussis in the primary analysis were prescribed antibiotics for cough 1 month before their pertussis diagnosis. The most prescribed antibiotics were macrolides (83%) and broad-spectrum penicillin (24%), followed by tetracyclines (5%), cephalosporins (4%), and trimethoprim combinations (1%) (Table 5).

**Table 5:** Distribution of antibiotics prescribed within 1 month before pertussis diagnosis in persons ≥50 years of age, Australia*

Category	No. pertussis cases	Antibiotic prescription			Antibiotic category				
		No	Yes		Broad-spectrum penicillin	Cephalosporins	Macrolides	Tetracyclines	Trimethoprim combinations
All pertussis patients									
Overall	992	740 (75)	252 (25)		61 (24)	11 (4)	209 (83)	12 (5)	2 (1)
Age group, y									
50–64	598	444 (74)	154 (26)		33 (21)	4 (3)	130 (84)	6 (4)	1 (1)
65–74	260	190 (73)	70 (27)		17 (24)	6 (9)	60 (86)	1 (1)	0 (0)
75–84	95	76 (80)	19 (20)		8 (42)	1 (5)	11 (58)	5 (26)	0 (0)
>85	39	30 (77)	9 (23)		3 (33)	0 (0)	8 (89)	0 (0)	1 (11)
Sex									
F	658	497 (76)	161 (24)		36 (22)	9 (6)	134 (83)	7 (4)	2 (1)
M	332	242 (73)	90 (27)		25 (28)	2 (2)	74 (82)	5 (6)	0 (0)
Pertussis patients with asthma									
Overall	117	75 (64)	42 (36)		14 (33)	7 (17)	34 (81)	0	NA
Age group, y									
50–64	72	47 (65)	25 (35)		7 (28)	3 (12)	21 (84)	0	
65–74	38	21 (14)	17 (86)		6 (35)	4 (24)	14 (82)	0	NA
75–84	7	6 (86)	1 (14)		1 (100)	0	0	0	
>85	0	0	0		0	0	0	0	
Sex									
F	86	55 (64)	31 (36)		10 (32)	7 (23)	26 (84)	0	NA
M	31	20 (65)	11 (35)		4 (36)	0	8 (73)	0	
Pertussis patients with COPD									
Overall	185	139 (75)	46 (25)		17 (37)	2 (4)	38 (83)	3 (7)	NA
Age group, y									
50–64	81	61 (75)	20 (25)		7 (35)	0	16 (80)	1 (5)	
65–74	60	44 (73)	16 (27)		6 (38)	2 (13)	14 (88)	0	NA
75–84	29	22 (76)	7 (24)		2 (29)	0	6 (86)	2 (29)	
>85	15	11 (73)	4 (27)		2 (50)	0	3 (75)	0	
Sex									
F	143	110 (77)	33 (23)		8 (24)	2 (6)	29 (88)	2 (6)	NA
M	42	29 (69)	13 (31)		9 (69)	0	9 (69)	1 (8)	
Pertussis patients without asthma/COPD									
Overall	709	534 (75)	175 (25)		34 (19)	4 (2)	147 (84)	9 (5)	2 (1)
Age group, y									
50–64	452	337 (75)	115 (25)		21 (18)	1 (1)	99 (86)	5 (4)	1 (1)
65–74	173	129 (75)	44 (25)		7 (16)	2 (5)	38 (86)	1 (2)	0
75–84	60	49 (82)	11 (18)		5 (45)	1 (9)	5 (45)	3 (27)	0
>85	24	19 (79)	5 (21)		1 (20)	0	5 (100)	0	1 (20)
Sex									
F	443	337 (76)	106 (24)		21 (20)	2 (2)	87 (82)	5 (5)	2 (2)
M	264	196 (74)	68 (26)		13 (19)	2 (3)	59 (87)	4 (6)	0

*Values are no. (%) except as indicated. Percentages were calculated within each row. Because some patients may have received combination of antibiotics, the sum of number of patients in antibiotics category do not equal to total number of pertussis cases in each row. Likewise, percentages do not sum up to 100%. COPD, chronic obstructive pulmonary disease; NA, not applicable.

## Discussion

By using the IQVIA GP EMR database, we estimated IRs and risk factors for clinically diagnosed pertussis among Australians ≥50 years, including those with COPD or asthma (Appendix Figure 5). IRs were highest among patients 50–64 years of age and were 2 times higher in women than in men. Furthermore, compared with the overall study population, IRs in patients with COPD were ≈3–4 times higher and in patients with asthma were ≈5–8 times higher.

In a systematic literature review reporting results from 85 publications, IRs in older adults broadly ranged 2–16,670/100,000 population, depending on the definition of pertussis and study methodology (*4*). Our findings are largely consistent with previously published global studies and studies in Australia, also reporting higher pertussis IRs in women compared with men (*11*,*13*,*14*). The higher IRs in women may be attributed to healthcare-seeking behavior, increased exposure to children and elderly persons infected with pertussis through caretaker roles, and biological and immune response differences (*14*).

For the extended definition of pertussis in the sensitivity analysis, we noted pertussis IRs to be higher than those in the primary analysis, likely attributable in part to the inclusion of nonpertussis seasonal respiratory diseases. In patients with COPD or asthma, we found IRs in the sensitivity analysis to be >3 times higher than those observed in the primary analysis, although the overall trends were consistent.

Trends observed in IRs were largely consistent between GP EMR data and NNDSS data, aligning with the known cyclic trend of pertussis epidemics in Australia. Contemporary data suggest that pertussis outbreaks occur periodically every 3 to 4 years (*5*,*15*); the 2 most recent epidemics of pertussis in Australia peaked in 2011 and 2015 (*16*). Evidence of seasonal fluctuations in pertussis incidence has been noted in other countries as well (*17*–*21*). There are conflicting findings in the context of Australia; several studies report higher incidence peaks in spring and summer months (November–January) and others report such peaks in winter months (June–September) (*22*–*24*). Pertussis was also found to be a common pathogen in infants hospitalized for acute lower respiratory tract infection, including pertussis co-infection, during winter in the Netherlands (*25*). It is therefore likely that the incidence spikes of pertussis observed in our study were confounded by circulating influenza viruses or respiratory syncytial virus outbreaks because of the the non–laboratory-confirmed definition of pertussis cases.

Conditional multivariable regression analysis showed that prior use of antibiotics within 1 month and concurrent asthma and COPD were predictors of pertussis diagnosis. Because only 24% of patients with pertussis in this study were tested for pertussis, antibiotics prescribed for concurrent asthma and COPD may have influenced prediction of pertussis diagnosis.

Patients with a history of influenza vaccination had a 62% lower risk for pertussis diagnosis than those without that history. A post-hoc analysis from a clinical trial among pregnant women suggested that influenza vaccination may have a positive effect on rates of pertussis infection and called for further investigation into the possible mechanisms in the upper respiratory tract that can lead to the synergy between the 2 pathogens (*26*). Influenza-vaccinated persons have a lower risk for influenza-related worsening of other respiratory diseases (*27*); thus, fewer potentially misdiagnosed pertussis cases may contribute to artifacts rather than true association between influenza vaccination history and pertussis diagnosis. Given the low pertussis testing rates in this study, these findings should be treated with caution. Notably, the Australian government offers free influenza vaccines through the National Immunisation Program for seniors ≥65 years of age, which may have contributed to a higher influenza vaccination rate in the study population. In addition, patients in better health or who are health-conscious are more likely to get vaccinated (i.e., healthy vaccinee bias) and seek health care when experiencing respiratory symptoms (*28*).

The risk for pertussis diagnosis in patients with underlying COPD and asthma was previously reported to be higher than for patients with pertussis but without those conditions (*29*). Contemporary data also suggest that patients with COPD and asthma have a higher pertussis incidence, risk for pertussis infection, and greater subsequent disease burden than patients with pertussis but without such concurrent conditions (*9*,*29*–*31*). Furthermore, asthma, COPD, and other chronic inflammatory pulmonary diseases can be exacerbated by *B. pertussis* infection (*29*). Increased risk for pertussis in patients with COPD or asthma may be explained by potential adverse effects of inhaled or systemic corticosteroids, altered airway architecture, impairment of innate and acquired immunity, waning humoral immunity over time, and influence of concurrent conditions on immunogenicity (*30*).

In this study, women, older age groups, and those with a history of influenza vaccination were associated with a higher median number of GP visits in a year, likely resulting in a higher chance of pertussis being recognized or diagnosed. Those observations might be due to women and older adults (who may have other concurrent conditions) visiting the GP more often (*32*–*34*). The higher median number of GP visits in persons with a history of influenza vaccination versus without suggests that those persons are more health conscious (i.e., healthy vaccinee bias) or had other conditions that required more GP visits. Among all patients diagnosed with pertussis, antibiotic prescriptions within 1 month before pertussis diagnosis were more common for those with COPD or asthma, men, and younger age groups (50–74 years); macrolides and penicillin were the most prescribed. Of note, persistent cough is a criterion for asthma and COPD exacerbation according to Australia guidelines (*35*,*36*), possibly explaining why antibiotics were more commonly prescribed and why pertussis may not have been recognized or diagnosed earlier in these patients. Because macrolides are the preferred treatment for pertussis, an adequate dosage and treatment duration may reduce likelihood of pertussis diagnosis (*37*), which may have skewed the estimated pertussis IRs in the populations described in this study, particularly among patients with COPD or asthma.

Because of differing case definitions in this study and the lack of routine laboratory testing in the primary care setting, the pertussis IRs in Australians ≥50 years may be higher than those reported to the NNDSS. NNDSS data rely on GP clinical, laboratory, or empirical diagnoses to identify confirmed and probable cases of pertussis. The surveillance case definition for pertussis requires confirmed cases to have either laboratory-definitive evidence or laboratory-suggestive evidence and clinical evidence, whereas probable cases require clinical and epidemiologic evidence (*38*). In our study, we identified cases based on clinical diagnosis, and only a small proportion of patients had pertussis-related diagnostic laboratory tests performed and documented in the EMR. Therefore, the approach we used in this study captured more cases that did not meet the NNDSS case definition of pertussis. Estimating the true impact of pertussis vaccination is not feasible, given the low vaccination rate and underdocumentation of vaccination history in the EMR database. Previous studies have shown that pertussis-related hospitalizations, healthcare burden, concurrent conditions, and risk for death increase with age (*4*,*11*,*39*). However, because the GP EMR database is based in the primary care setting, it is not representative of all Australians ≥50 years and does not allow further evaluation of pertussis severity and its association with hospitalization, healthcare burden, and mortality. Given the known underdiagnosis and underreporting of pertussis, our incidence and risk factor analysis can still provide useful guidance related to pertussis prevention in the primary care setting. Nevertheless, it will be important to further validate the findings of this study by using a more specific and validated case definition of pertussis cases.

From a public health standpoint, this study highlights the importance of pertussis prevention for Australians ≥50 years, especially among those with COPD or asthma, thus improving community awareness and uptake of immunization at age 50 and 65 years, which is currently suboptimal (*40*,*41*). An effective vaccination strategy is necessary to reduce complications and increased healthcare costs associated with pertussis among patients with COPD or asthma (*4*,*29*). For patients with COPD or asthma who have unrecognized or undiagnosed pertussis, inappropriately prescribed antibiotics may empirically treat pathogens causing other respiratory illnesses but delay the diagnosis and control of pertussis. Conversely, timely pertussis vaccinations can protect vaccinated persons against pertussis development and prevent community-level transmission. Other strategies include increasing pertussis testing by GPs to improve ascertainment of cases and ensure timely treatment (*40*).

Future studies might consider the effect of policy interventions on pertussis vaccination behavior, underuse of pertussis vaccines, and cost-effectiveness of potential new models of pertussis immunization programs to address policy gaps in the present program. In summary, a sizeable burden of pertussis in older adults in Australia has a greater impact on persons with COPD or asthma. This study highlights the importance of pertussis prevention for Australians ≥50 years of age.

Appendix Additional information for population-based study of pertussis incidence and risk factors among persons >50 years of age, Australia.
