# Supplementary material for: Population-Based Study of Pertussis Incidence and Risk Factors among Persons >50 Years of Age, Australia
Source: Emerg Infect Dis. 2024 Jan;30(1):105–15. doi: 10.3201/eid3001.230261 (PMC10756356; doi:10.3201/eid3001.230261)
Supplement: Appendix — Additional information for population-based study of pertussis incidence and risk factors among persons >50 years of age, Australia. [file 23-0261-Techapp-s1.pdf]

Article DOI: http://doi.org/10.3201/eid3001.230261

EID cannot ensure accessibility for supplementary materials supplied by authors. Readers who have difficulty accessing supplementary content should contact the authors for assistance.

Population-Based Study of Pertussis Incidence and Risk Factors among Persons ≥50 Years of Age, Australia

Appendix

Appendix Table 1. Search terms used for the case definitions of pertussis, COPD, and asthma

	Search Term Categories		
Condition	Inclusion	Exclusion	Additional terms for sensitivity analysis
Pertussis	%whoop%in%	%immun%	%post%tussive%vomit%
	%pertus%is%	%vaccin%	%post%tussive%emesis%
	%pert%vacc%		paroxy%cough
	%pert%ssis%		night%cough
	%pert%ssus%		cough%night
	%whoop%cough%		
	%cough%whoop%		
COPD and Asthma	Diagnosis Search Terms	Product Search Terms	
	%asthma%	ACLIDINIUM%	NEDOCROMIL%
	%COAD%	AERON%	NUELIN%
	%Chronic%Airways%	AIROMIR%	OLODATEROL%
	'%Ã%HMA%	ALVESCO%	OMALIZUMAB%
	%Emphysema%	ANORO%ELLIPTA%	ONBREZ%BREEZHALER%
	%COPD%	APOVEN%	OPTROL%
	%a%hma%	ASMOL%	OXIS%
	%Chronic%Pulmonary%	ATROVENT%	PULMICORT%
	%asmatic%	BECLOMETHASONE%	QVAR%
		BREO%ELLIPTA%	RESPAX%
		BRETARIS%GENUAIR%	RESPIKAST%
		BRICANYL%	RESPIMAT%
		BRIMICA%GENUAIR%	RESPOLIN%
		BUDESONIDE%	SALBUTAMOL%
		BUTAMOL%	SALMETEROL%
		CICLESONIDE%	SEEBRI%
		EFORMOTEROL%	SERETIDE%
		EPAQ%INHALER%	SEREVENT%
		FLIXOTIDE%	SINGULAIR%
		FLUTICASONE%	SODIUM%CROMOGLYCATE%
		FLUTIFORM%	SPIOLTO%
		FORADILE%	SPIRIVA%
		GLYCOPYRRONIUM%	SYMBICORT%
		INCRUSE ELLIPTA%	T%LUKAST%
		INDACATEROL%	TERBUTALINE%
		INTAL%	THEO-DUR%
		IPRATRIN%	THEOPHYLLINE%
		IPRATROPIUM%	TILADE%
		IPRAVENT%	TIOTROPIUM%
		LUKAIR%	ULTIBRO%BREEZHALER%
		MONTAIR%	UMECLIDINIUM%
		MONTELUKAST%	VENTOLIN%
			XOLAIR%

COPD: chronic obstructive pulmonary disease.

Appendix Table 2. Extended case definition of pertussis in the sensitivity analysis, including those who had not been documented with pertussis/whooping cough in the diagnosis field, but who met the criteria below.

Criteria for inclusion in sensitivity analysis
Patients who had cough (non-paroxysmal) for more than 2 weeks not treated with antibiotics
Patients who had paroxysmal cough, post-tussive vomiting or night cough for more than 2 weeks treated with antibiotics (*1*,*2*)
Patients who had cough for more than 2 weeks AND prescribed clarithromycin (up to 500 mg twice daily for 7 d) OR azithromycin (up to 500 mg once daily for 3 to 5 d) OR erythromycin (up to 250 mg four times daily for 7 d) OR trimethoprim with sulfamethoxazole (160/800 mg twice daily for 7 d) (*3*) i.e., those treated according to the recommended treatment guidelines for pertussis disease
Patients who had cough for more than 2 weeks AND prescribed clarithromycin (up to 500 mg twice daily for 14 to 21 d) OR azithromycin (up to 500 mg once daily for at least 5 d) OR erythromycin 500 mg four times daily for 14 to 21 d AND Augmentin 1 tablet twice daily for 7 d (*4*–*6*), i.e., those treated as atypical pneumonia
Patients who had persistent cough without diagnosis of pertussis/whooping cough AND laboratory examinations performed. These tests include bacterial culture, polymerase chain reaction or immunofluorescence assays of nasopharyngeal swab or aspirate samples, serologic testing to detect rises in immunoglobulin (Ig) A or IgG titers to *B. pertussis* antigens, lymphocyte count (raised counts are a non-specific indicator of infection)
Patients who had persistent cough and presented more than once to the GP within 4 consecutive months (*7*)

Ig: immunoglobin; GP: general practitioner.

Appendix Table 3. Baseline patient characteristics of pertussis patients aged ≥50 y included in the sensitivity analysis by sex during the study period.

Characteristics*	Overall population (n = 2543)	Males (n = 1016)	Females (n = 1522)	p-value
Age, years [mean, (95% CI)]	65.3 (65.3–65.3)	65.3 (64.7–65.9)	65.3 (64.9–65.8)	0.93
Age, years [median (IQR)]	66 (14)	65 (15)	65 (15)	0.89
Smoking status, n (%)				
Never	1,191 (47%)	437 (43%)	753 (49%)	<0.001
Past	616 (24%)	295 (29%)	319 (21%)	
Current	192 (8%)	91 (9%)	101 (7%)	
Unknown	544 (21%)	193 (19%)	349 (23%)	
Alcohol consumption/day, n (%)				
None	1,214 (48%)	418 (41%)	793 (52%)	<0.001
≤1 unit	519 (20%)	217 (21%)	302 (20%)	
>1 unit	239 (9%)	152 (15%)	87 (6%)	
Unknown	571 (22%)	229 (23%)	340 (22%)	
History of pertussis immunization, n (%)	136 (5%)	59 (6%)	77 (5%)	0.36
History of flu vaccination, n (%)	507 (20%)	230 (23%)	276 (19%)	0.01
History of pneumococcal vaccination, n (%)	120 (5%)	50 (5%)	70 (5%)	0.29
Residence, n (%)				
New South Wales	526 (21%)	197 (19%)	328 (22%)	0.20
Victoria	549 (22%)	226 (22%)	322 (21%)	0.54
Queensland	358 (14%)	150 (15%)	208 (14%)	0.47
Western Australia	410 (16%)	162 (16%)	247 (16%)	0.89
South Australia	79 (3%)	32 (3%)	47 (3%)	0.42
Tasmania	193 (8%)	67 (7%)	125 (8%)	0.15
Australian Capital Territory	362 (14%)	157 (15%)	204 (13%)	0.16
Northern Territory	66 (3%)	25 (2%)	41 (3%)	0.81
Concurrent conditions, n (%)				
COPD	513 (20%)	185 (18%)	328 (22%)	0.06
Asthma	274 (11%)	93 (9%)	180 (12%)	0.01
CVD	1,008 (40%)	431 (42%)	576 (38%)	0.01
Heart failure	47 (2%)	20 (2%)	27 (2%)	0.13
Diabetes mellitus	351 (14%)	170 (17%)	181 (12%)	<0.001
Stroke	50 (2%)	25 (2%)	25 (2%)	0.04
Chronic kidney disease	79 (3%)	40 (4%)	39 (3%)	0.03
Cancer (active or remission)	370 (15%)	155 (15%)	215 (14%)	0.72

*Variables were defined as those at baseline, i.e., restricted to those recorded during the study period or until the time of pertussis diagnosis. The history of pertussis immunization was defined as the presence of pertussis immunization record throughout the study period, as recorded in the GP EMR database. The history of CVD, chronic kidney disease, and cancer were defined as those at baseline and were restricted to those recorded during the study period, or until the time of pertussis diagnosis, as applicable; CI: confidence interval; COPD: chronic obstructive pulmonary disease; CVD: cardiovascular disease (except heart failure); IQR: interquartile range. To compare the groups, Pearson's chi-square or Fisher's exact test were used on categorical variables. Student's t-test/Wilcoxon/Kruskal Wallis tests were applied on continuous variables (as appropriate). There were significant differences in smoking status, alcohol consumption, history of flu vaccination, asthma, CVD, diabetes, stroke, and chronic kidney disease between males and females.

Appendix Table 4. Baseline characteristics of pertussis patients aged ≥50 y included in the sensitivity analysis by age groups between 2015 and 2019.

	Age groups, years				
Characteristics*	50–64 (n = 1283)	65–74 (n = 815)	75–84 (n = 347)	≥85 (n = 98)	p-value
Sex, n (%)					
M	508 (40%)	332 (41%)	135 (39%)	41 (42%)	0.86
F	771 (60%)	482 (59%)	212 (61%)	57 (58%)	
Smoking status, n (%)					
Never	589 (46%)	378 (46%)	177 (51%)	47 (48%)	<0.001
Past	251 (20%)	233 (29%)	100 (29%)	32 (33%)	
Current	131 (10%)	45 (6%)	15 (4%)	1 (1%)	
Unknown	312 (24%)	159 (20%)	55 (16%)	18 (18%)	
Alcohol consumption/day, n (%)					
None	614 (48%)	394 (48%)	149 (43%)	57 (58%)	<0.001
≤1 unit	212 (17%)	178 (22%)	108 (31%)	21 (21%)	
>1 unit	136 (11%)	75 (9%)	25 (7%)	3 (3%)	
Unknown	321 (25%)	168 (21%)	65 (19%)	17 (17%)	
History of pertussis immunization, n (%)	60 (5%)	56 (7%)	15 (4%)	5 (5%)	0.24
History of flu vaccination, n (%)	109 (8%)	217 (27%)	150 (43%)	31 (32%)	<0.001
History of pneumococcal vaccination, n (%)	10 (1%)	77 (9%)	29 (8%)	4 (4%)	<0.001
Residence, n (%)					
New South Wales	265 (21%)	171 (21%)	69 (20%)	21 (21%)	0.98
Victoria	304 (24%)	163 (20%)	64 (18%)	18 (18%)	0.07
Queensland	163 (13%)	122 (15%)	58 (17%)	15 (15%)	0.20
Western Australia	195 (15%)	144 (18%)	57 (16%)	14 (14%)	0.47
South Australia	32 (2%)	32 (4%)	10 (3%)	5 (5%)	0.16
Tasmania	99 (8%)	56 (7%)	30 (9%)	8 (8%)	0.75
Australian Capital Territory	175 (14%)	113 (14%)	58 (17%)	16 (16%)	0.46
Northern Territory	50 (4%)	14 (2%)	1 (0%)	1 (1%)	<0.001
Concurrent conditions, n (%)					
COPD	179 (14%)	199 (24%)	102 (29%)	33 (34%)	<0.001
Asthma	132 (10%)	90 (11%)	40 (12%)	12 (12%)	0.85
CVD	392 (31%)	379 (47%)	182 (52%)	55 (56%)	<0.001
Heart failure	10 (1%)	12 (1%)	14 (4%)	11 (11%)	<0.001
Diabetes mellitus	129 (10%)	127 (16%)	79 (23%)	16 (16%)	<0.001
Stroke	8 (1%)	17 (2%)	17 (5%)	8 (8%)	<0.001
Chronic kidney disease	16 (1%)	26 (3%)	28 (8%)	9 (9%)	<0.001
Cancer (active or remission)	130 (10%)	138 (17%)	78 (22%)	24 (24%)	<0.001

*Variables were defined as those at baseline, i.e., restricted to those recorded during the study period or until the time of pertussis diagnosis. The history of pertussis immunization was defined as the presence of pertussis immunization record throughout the study period, as recorded in the GP EMR database. The history of CVD, chronic kidney disease, and cancer were defined as those at baseline and were restricted to those recorded during the study period, or until the time of pertussis diagnosis, as applicable; COPD: chronic obstructive pulmonary disease; CVD: cardiovascular disease (except heart failure). To compare the groups, Pearson's chi-square or Fisher's exact test were used on categorical variables. Student's t-test/Wilcoxon/Kruskal Wallis tests were applied on continuous variables (as appropriate). There were significant differences in smoking status, alcohol consumption, history of flu and pneumococcal vaccinations, state of residence (Northern Territory), COPD, CVD, heart failure, diabetes, stroke, chronic kidney disease, and cancer between the age groups.

Appendix Table 5. Incidence rates of pertussis in patients aged ≥50 y by sex and age groups between 2015 and 2019 in the overall study population (primary analysis).

	Year					
Category	2015	2016	2017	2018	2019	p-trend
As observed						
Total population in GP EMR, N	415,210	367,472	299,552	281,434	252,227	
Sex, n (incidence, 95% CI)						
M	137 (70.56, 64.33–76.78)	77 (45, 38.77–51.22)	73 (52.31, 46.08–58.53)	56 (42.6, 36.38–48.83)	49 (41.6, 35.38–47.83)	0.09
F	243 (110.53, 102.34–118.73)	173 (88.86, 80.67–97.06)	146 (92.09, 83.9–100.29)	105 (70.72, 62.53–78.91)	99 (74.5, 66.31–82.7)	0.22
Age groups, years, n (incidence, 95% CI)						
50–64	239 (105.91, 98.06–113.77)	153 (77.29, 69.44–85.15)	132 (86.7, 78.85–94.56)	91 (64.46, 56.6–72.31)	83 (67.07, 59.22–74.93)	0.22
65–74	95	67	62	41	45	0.22

Category	Year 2015	2016	2017	2018	2019	p-trend
	(86.21, 78.95–93.47)	(68.42, 61.16–75.68)	(74.2, 66.94–81.46)	(51.6, 44.34–58.86)	(62.45, 55.19–69.71)	
75–84	32 (55.32, 49.61–61.03)	21 (41.4, 35.69–47.11)	19 (42.23, 36.52–47.94)	21 (48.51, 42.8–54.22)	16 (39.43, 33.72–45.14)	0.81
≥85	15 (63.91, 58.08–69.73)	10 (47.9, 42.07–53.72)	6 (31.98, 26.15–37.81)	9 (51.39, 45.56–57.22)	4 (25.24, 19.42–31.07)	0.22
Projected to national level						
Total Australian population, N*	7,842,028	8,001,533	8,165,022	8,330,359	8,506,287	
Sex, (n, 95% CI)						
Males	2,657 (2,423–2,891)	1,728 (1,489–1,967)	2,048 (1,805–2,292)	1,701 (1,453–1,950)	1,695 (1,442–1,949)	
Females	4,506 (4,172–4,840)	3,698 (3,357–4,039)	3,913 (3,565–4,261)	3,067 (2,712–3,423)	3,301 (2,938–3,664)	
Age groups, years, (n, 95% CI)						
50–64	4,541 (4,204–4,878)	3,346 (3,006–3,686)	3,793 (3,449–4,136)	2,847 (2,500–3,194)	2,997 (2,646–3,348)	
65–74	1,730 (1,584–1,876)	1,425 (1,274–1,577)	1,597 (1,440–1,753)	1,148 (986–1,309)	1,426 (1,260–1,591)	
75–84	597 (535–658)	458 (395–521)	484 (419–550)	575 (508–643)	489 (418–560)	
≥85	300 (272–327)	231 (203–259)	158 (129–186)	259 (229–288)	130 (100–160)	

CI: confidence interval; EMR: electronic medical records; GP: general practitioner. Incidence rates are reported per 100,000 persons. In the projected to national level section, the numbers refer to the estimated number of pertussis patients in Australia based on the calculated incidence rates. Data in 2015 were projected to a 12-mo period because a run-in period/landmark was applied to rule out prevalent pertussis cases carried forward from the previous year. Data for 2016–2019 are as observed. Source: (*8*).

Appendix Table 6. Incidence rates of pertussis in patients aged ≥50 y with COPD by sex and age groups between 2015 and 2019 (primary analysis).

Category	Year 2015	2016	2017	2018	2019	p-trend
As observed						
Total COPD population in GP EMR, N	20,204	20,092	17,874	16,212	12,870	
Overall population, n (Incidence, 95% CI)	62 (304.4, 290.7–318.1)	51 (253.8, 240.1–267.6)	40 (223.8, 210.1–237.5)	38 (234.4, 220.7–248.1)	25 (194.3, 180.5–207.9)	
Sex, n (incidence, 95% CI)						
M	21 (187.3, 178.2–196.4)	9 (79.2, 70.1–88.3)	10 (99.0, 89.9–108.1)	9 (98.9, 89.9–108.0)	5 (68.3, 59.2–77.4)	0.22
F	41 (451.6, 433.1–469.9)	42 (482.3, 463.9–500.7)	30 (387.1, 368.6–405.5)	29 (409.3, 390.8–427.7)	20 (361.9, 343.4–380.3)	0.22
Age groups, years, n (incidence, 95% CI)						
50–64	35 (483.5, 467.4–499.7)	19 (266.6, 250.5–282.8)	17 (281.5, 265.3–297.7)	17 (315.9, 299.8–332.2)	11 (267.9, 251.7–284.1)	0.81
65–74	20 (303.2, 289.2–317.2)	19 (298.6, 284.6–312.6)	14 (248.5, 234.5–262.5)	8 (153.9, 139.9–167.9)	9 (217.5, 203.5–231.5)	0.09
75–84	6 (129.7, 118.7–140.7)	8 (173.5, 162.5–184.5)	6 (137.6, 126.6–148.6)	8 (199.6, 188.6–210.6)	4 (120.1, 109.0–131.1)	0.61
≥85	2 (74.6, 62.4–86.7)	5 (251.1, 238.9–263.3)	3 (163.1, 150.9–175.3)	5 (307.7, 295.5–319.8)	1 (77.3, 65.1–89.4)	0.81
Projected to national level						
Total COPD Australian population, N*	429,805	438,548	447,510	456,572	466,216	
Sex, (n, 95% CI)						
M	385 (366–404)	166 (147–185)	212 (192–231)	216 (196–236)	152 (132–172)	
F	1,012 (971–1054)	1,104 (1062–1146)	904 (861–947)	976 (932–1020)	882 (837–927)	
Age groups, years, (n, 95% CI)						
50–64	746 (721–771)	416 (390–441)	443 (418–469)	502 (477–528)	431 (405–457)	

Category	Year 2015	2016	2017	2018	2019	p-trend
65–74	432 (412–452)	442 (421–462)	380 (358–401)	243 (221–265)	353 (330–375)	
75–84	92 (85–100)	127 (119–135)	104 (96–112)	156 (148–165)	98 (89–107)	
≥85	23 (19–27)	80 (76–84)	53 (49–57)	102 (98–106)	26 (22–30)	

CI: confidence interval; COPD: chronic obstructive pulmonary disease; EMR: electronic medical records; GP: general practitioner. Incidence rates are reported per 100,000 persons. In the projected to national level section, the numbers refer to the estimated number of pertussis patients in Australia based on the calculated incidence rates. Data in 2015 were projected to 12-mo period because a run-in period/landmark was applied to rule out prevalent pertussis cases carried forward from the previous year. Data for 2016–2019 are as observed. Sources: (*9*,*10*).

Appendix Table 7. Incidence rates of pertussis in patients aged ≥50 y with asthma by sex and age groups between 2015 and 2019.

Category	Year 2015	2016	2017	2018	2019	p-trend
As observed						
Total asthma population in GP EMR, N	7,928	7,433	6,188	5,474	4,246	
Overall population, n (Incidence, 95% CI)	38 (473.0, 454.0–492.0)	35 (470.9, 451.9–489.9)	27 (436.3, 417.3–455.3)	24 (438.4, 419.4–457.4)	16 (376.8, 357.8–395.8)	
Sex, n (incidence, 95% CI)						
M	11 (213.2, 200.8–225.6)	11 (239.6, 227.1–251.9)	4 (103.8, 91.4–116.2)	6 (176.6, 164.2–189.0)	6 (224.6, 212.1–236.9)	0.81
F	27 (903.0, 873.2–932.9)	24 (849.3, 819.4–879.1)	23 (991.4, 961.5–1,021.2)	18 (871.7, 841.8–901.5)	10 (641.9, 612.0–671.7)	0.46
Age groups, years, n (incidence, 95% CI)						
50–64	21 (508.6, 486.4–530.8)	19 (490.6, 468.4–512.7)	15 (488.6, 466.4–510.8)	17 (636.5, 614.3–658.6)	12 (599.1, 576.9–621.3)	0.81
65–74	17 (743.9, 721.7–766.2)	12 (567.1, 544.9–589.3)	11 (597.5, 575.3–619.7)	6 (378.6, 356.3–400.8)	3 (230.9, 208.7–253.2)	0.09
75–84	NA	4 (385.7, 372.6–398.8)	1 (107.6, 94.5–120.8)	1 (110.9, 97.9–124.1)	1 (142.5, 129.3–155.6)	
≥85	NA	NA	NA	NA	NA	
Projected to national level						
Total asthma Australian population, N*	948,708	968,084	987,938	1,008,024	1,029,393	
Sex, (n, 95% CI)						
M	829 (780–877)	949 (900–999)	420 (369–470)	728 (676–779)	944 (892–997)	
F	5,058 (4,890–5,225)	4,856 (4,685–5,027)	5,787 (5,613–5,962)	5,194 (5,017–5,372)	3,908 (3,726–4,090)	
Age groups, years, (n, 95% CI)						
50–64	2,715 (2,597–2,833)	2,644 (2,525–2,764)	2,661 (2,540–2,781)	3,500 (3,378–3,622)	3,333 (3,209–3,456)	
65–74	1,881 (1,825–1,937)	1,489 (1,430–1,547)	1,620 (1,560–1,680)	1,061 (998–1,123)	664 (600–728)	
75–84	NA	491 (474–507)	142 (125–159)	151 (133–169)	203 (184–222)	
≥85	NA	NA	NA	NA	NA	

CI: confidence interval; EMR: electronic medical records; GP: general practitioner; NA: not applicable. Incidence rates are reported per 100,000 persons. In the projected to national level section, the numbers refer to the estimated number of pertussis patients in Australia based on the calculated incidence rates. Data in 2015 were projected to 12-mo period because a run-in period/landmark was applied to rule out prevalent pertussis cases carried forward from the previous year. Data for 2016–2019 are as observed. Sources: (*9*,*10*).

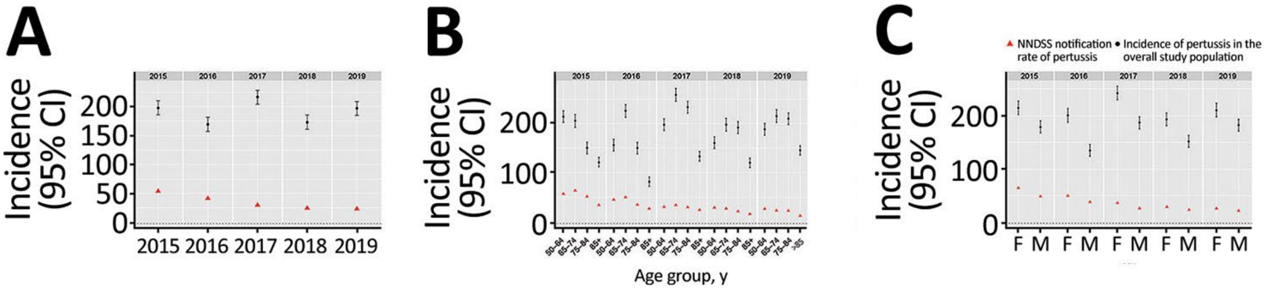

Appendix Figure 1. Annual incidence of pertussis in the overall study population (sensitivity analysis) (a), by age group (b), and by sex (c). EMR: electronic medical records; GP: general practitioner; NNDSS: National Notifiable Diseases Surveillance System. Error bars indicate 95% confidence intervals. Incidence rates are reported per 100,000 persons. Both NNDSS and GP EMR data consist of persons aged ≥50 years. Data in 2015 were projected to 12-month period because a run-in period/landmark was applied to rule out prevalent pertussis cases carried forward from the previous year. Data for 2016–2019 are as observed.

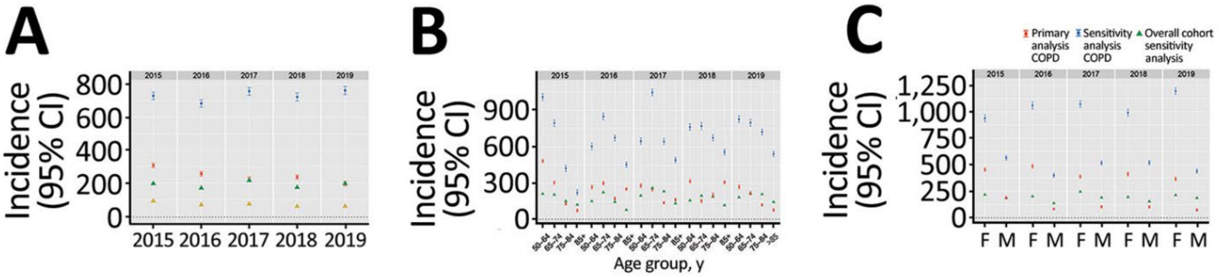

Appendix Figure 2. Annual incidence of pertussis in patients in the overall population (sensitivity analysis) with COPD (a), by age group (b), and by sex (c). COPD: chronic obstructive pulmonary disease; EMR: electronic medical record; GP: general practitioner; NNDSS: National Notifiable Diseases Surveillance System. Error bars indicate 95% confidence intervals. Incidence rates are reported per 100,000 persons. GP EMR data consist of people aged ≥50 years. Data in 2015 were projected to 12-month period because a run-in period/landmark was applied to rule out prevalent pertussis cases carried forward from the previous year. Data for 2016–2019 are as observed.

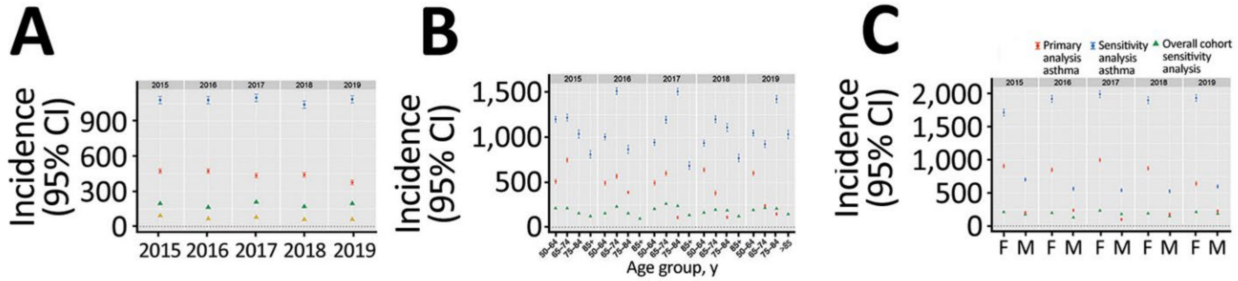

Appendix Figure 3. Annual incidence of pertussis in patients based on the overall population (sensitivity analysis) with asthma (a), by age group (b), and by sex (c). EMR: electronic medical records; GP: general practitioner; NNDSS: National Notifiable Diseases Surveillance System. Error bars indicate 95% confidence intervals. Incidence rates are reported per 100,000 persons. Both NNDSS and GP EMR data consist of people aged ≥50 years. Data in 2015 were projected to 12-month period because a run-in period/landmark was applied to rule out prevalent pertussis cases carried forward from the previous year. Data for 2016–2019 are as observed.

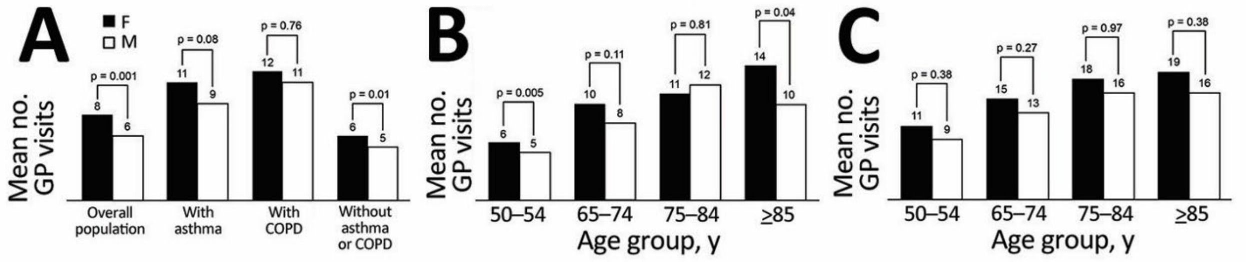

Appendix Figure 4. Median number of GP visits within 1 year before pertussis diagnosis in the overall population by sex and COPD or asthma status (a), in the overall population by sex and age groups (b), in the asthma and COPD population by sex and age groups (c). COPD: chronic obstructive pulmonary disease; GP: general practitioner. Quantitative variables were compared using a two-sample Student's t-test after checking equality of variance (Fisher's test) and normality (Shapiro–Wilk test); Wilcoxon, Mann–Whitney or Kruskal–Wallis tests or analysis of variance were used if equality of variance and normality were not confirmed. All tests are two-tailed, with a p value of 0.05 considered as statistically significant.

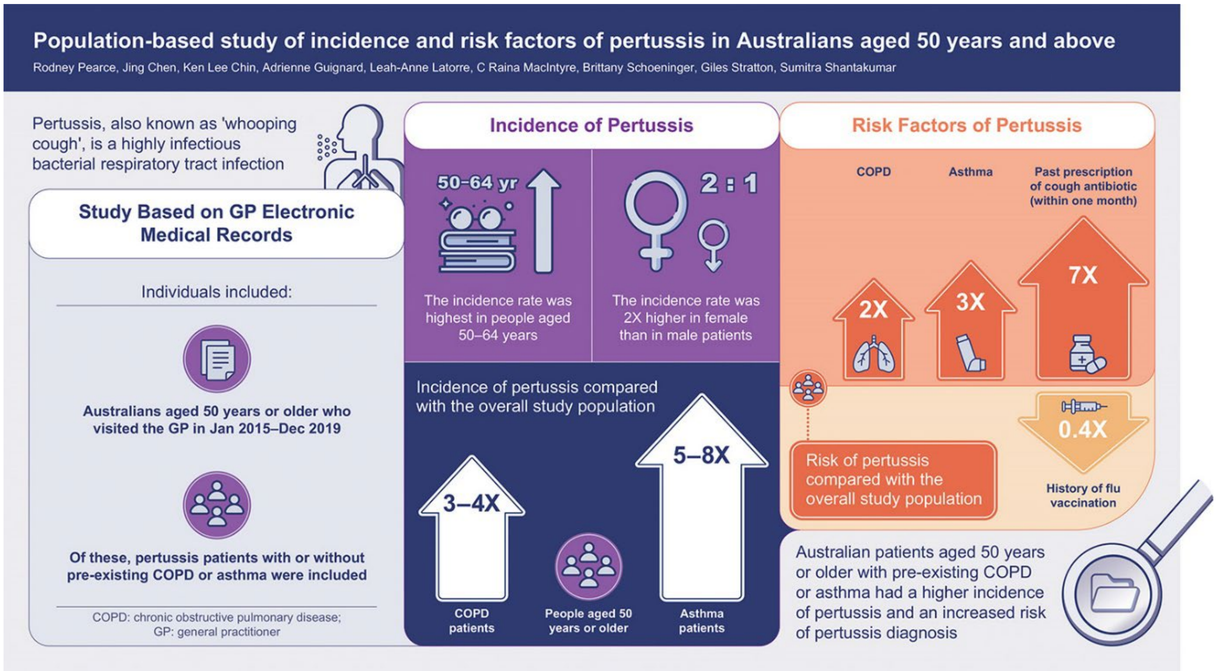

Appendix Figure 5. Visual description of study design and results for population-based study of incidence and risk factors of pertussis in Australians aged 50 years and above.

References

1. Cherry JD, Tan T, Wirsing von König CH, Forsyth KD, Thisyakorn U, Greenberg D, et al. Clinical definitions of pertussis: summary of a Global Pertussis Initiative roundtable meeting, February 2011. Clin Infect Dis. 2012;54:1756–64. PubMed https://doi.org/10.1093/cid/cis302

2. Moore A, Harnden A, Grant CC, Patel S, Irwin RS, Altman KW, et al.; CHEST Expert Cough Panel. Clinically diagnosing pertussis-associated cough in adults and children: CHEST guideline and expert panel report. Chest. 2019;155:147–54. PubMed https://doi.org/10.1016/j.chest.2018.09.027

3. Marchant J, Chang C. Managing pertussis in adults. Aust Prescr. 2009;32:36–8. https://doi.org/10.18773/austprescr.2009.019

4. Corrêa RA, Costa AN, Lundgren F, Michelin L, Figueiredo MR, Holanda M, et al. 2018 recommendations for the management of community acquired pneumonia. J Bras Pneumol. 2018;44:405–23. PubMed https://doi.org/10.1590/s1806-37562018000000130

5. Metlay JP, Waterer GW, Long AC, Anzueto A, Brozek J, Crothers K, et al. Diagnosis and treatment of adults with community-acquired pneumonia. An official clinical practice guideline of the American Thoracic Society and Infectious Diseases Society of America Am J Respir. Am J Respir Crit Care Med. 2019;200:e45–67. PubMed https://doi.org/10.1164/rccm.201908-1581ST

6. Nir-Paz R. Atypical pneumonia (non-COVID-19). BMJ Best Practice. 2020 Aug 11 [cited 2022 Aug 5]. https://bestpractice.bmj.com/topics/en-us/18

7. Kilgore PE, Salim AM, Zervos MJ, Schmitt HJ. Pertussis: microbiology, disease, treatment, and prevention. Clin Microbiol Rev. 2016;29:449–86. PubMed https://doi.org/10.1128/CMR.00083-15

8. Australian Bureau of Statistics. National, state and territory population. Canberra: ABS; 2020 [cited 2022 Dec 13]. https://www.abs.gov.au/statistics/people/population/national-state-and-territory-population/jun-2020

9. Australian Institute of Health and Welfare. Asthma. Canberra: Australian Institute of Health and Welfare; 2020 [cited 2022 Dec 13]. https://www.aihw.gov.au/reports/chronic-respiratory-conditions/asthma

10. Australian Bureau of Statistics. National Health Survey: first results. Canberra: ABS; 2017–18 [cited 2022 Dec 13]. https://www.abs.gov.au/statistics/health/health-conditions-and-risks/national-health-survey-first-results/latest-release
